# Single‐cell and spatial transcriptomics reveal apelin/APJ pathway's role in microvessel formation and tumour progression in hepatocellular carcinoma

**DOI:** 10.1111/jcmm.70152

**Published:** 2024-10-21

**Authors:** Yongfu Zhu, Pengcheng Zhang, Xingxing Huo, Yi Ling, Xiang Lv, Shengyou Lin, Hang Song

**Affiliations:** ^1^ The First Department of Oncology The First Affiliated Hospital of Anhui University of Chinese Medicine Hefei China; ^2^ Department of Dr. Hu Guojun Specialist Clinic The First Affiliated Hospital of Anhui University of Chinese Medicine Hefei China; ^3^ The First Affiliated Hospital of Zhejiang Chinese Medical University (Zheiiang Provincial Hospital of Chinese Medicine) Hangzhou China; ^4^ Experimental Center of Clinical Research, Scientific Research Department The First Affiliated Hospital of Anhui University of Chinese Medicine Hefei China; ^5^ The First Clinical Medical College Anhui University of Chinese Medicine Hefei Anhui China; ^6^ Department of Oncology Shanghai Traditional Chinese Medicine Hospital Shanghai China; ^7^ School of Integrated Chinese and Western Medicine Anhui University of Chinese Medicine Hefei China

**Keywords:** anti‐angiogenesis, apelin/APJ, hepatocellular carcinoma, spatial transcriptomics, tumour progression

## Abstract

The apelin receptor (APJ) is a key player in tumour angiogenesis, but its role in hepatocellular carcinoma (HCC) remains unclear. This study aims to elucidate the function of the apelin/APJ pathway in HCC using a multi‐omics approach and identify potential therapeutic biomarkers. Differentially expressed genes related to the apelin/APJ axis were identified from bulk transcriptomics to reveal HCC‐associated disparities. Single‐cell and spatial transcriptomics were used to localize and analyse the function of these genes. Machine learning models were constructed to predict outcomes based on apelin/APJ expression, and experimental validation was conducted to explore the pathway's impact on HCC angiogenesis. Single cell analysis revealed an overexpression of APJ/Aplin in the endothelium. The stemness of endothelial cell (EC) with high apelin/APJ was enhanced, as well as the expression of TGFb, oxidative stresses and PI3K/AKT pathway genes. Spatial transcriptomics confirmed that EC populations with high APJ scores were enriched within the tumour. Machine learning models showed high prognostic accuracy. High APJ expression was linked to worse outcomes (*p* = 0.001), and AUC values were high (1 year, 3 year, 5 year) (0.95, 0.97, 0.98). Immune suppression and non‐responsiveness of immune therapy were also seen in high‐risk groups. The experimental validation showed that silencing apelin reduced angiogenesis (*p*  < 0.05), endothelial proliferation, decreased expression of ANG2, KLF2, VEGFA and lower ERK1/2 phosphorylation. Apelin may serve as a potential therapeutic target in HCC, given its role in promoting tumour angiogenesis and poor patient outcomes.

## 1 INTRODUCTION

Liver cancer has been recognized as the fifth leading cause of malignancies among the males and the seventh among the females globally, with about one million new cases reported each year. Hepatocellular carcinoma (HCC) constitute about 80%–90% of primary liver cancers,^1^ respectively. Liver cancer often presents insidiously, with 70%–80% of cases diagnosed at an advanced, unresectable stage.^2^ Current treatment options, including transarterial chemoembolization, targeted therapies like sorafenib^3^ and lenvatinib,^4^ and combination treatments with anti‐angiogenic agents such as atezolizumab and bevacizumab,^5^ have become increasingly vital in managing HCC. However, therapeutic efficacy for advanced liver cancer remains limited.^6^ Angiogenesis, a critical factor for tumour growth and its metastasis, underscores the importance of anti‐angiogenic drugs in treating advanced HCC.^7^ Current vascular‐targeted agents affect both tumour and normal vasculature.^8^ Emphasizing the urgent need for anti‐angiogenic drugs capable of discerning between pathological and normal blood vessels.

The apelin receptor/the angiotensin‐like‐receptor 1 (APJ) is a G protein‐coupled receptor exhibits high expressions on vascular endothelial cells (ECs).^9^ Inhibition of apelin has been shown to significantly reduce hypoxia‐induced angiogenesis without affecting the VEGF/VEGF receptor 2 (VEGFR2) signalling pathway.^10^ While apelin/APJ expression is markedly upregulated in the endothelium during development, it is reduced in adult vascular endothelia.^11^ However, under pathological conditions such as tumour proliferation and myocardial infarction,^12^, ^13^ there is an increase in the expression of Apelin/APJ in ECs. In the context of malignancies, apelin/APJ has been found to facilitate the maturation of the tumour vasculature and exert a suppressive effect on immune cells.^14^, ^15^, ^16^ Additionally, studies have demonstrated that apelin/APJ promotes cell invasion and metastasis in gastric cancer, colorectal cancer and glioma. The utilization of inhibitors targeting apelin/APJ has led to obviously reduced metastatic foci size and enhanced tumour cell apoptosis.^17^, ^18^, ^19^ These results highlight the possibility of using the apelin/APJ as a highly valuable target for developing anti‐angiogenic therapies in cancer.

In our study, we localized apelin/APJ in HCC using single‐cell resolution and interpreted the spatial positioning of apelin/APJ within the tumour microenvironment (TME) through spatial transcriptomics. Leveraging machine learning algorithms, we identified key apelin/APJ‐associated genes and elucidated their roles within the HCC immune microenvironment. Finally, we conducted experiments based on our screening results to validate our conclusions.

## 2 METHODS

### 2.1 Dataset processing

We retrieved the single‐cell dataset GSE149614^20^ from the Gene Expression Omnibus (GEO) database, consisting of 10 patient samples including primary tumour (PT), portal vein tumour thrombus (PVTT), metastatic lymph node (MLN), as well as non‐tumour liver tissues. Genes with expression levels over 300 were retained, while mitochondrial gene expression was capped at 15%. Doublet cells were eliminated using the Scrublet^20^ package, and RNA contamination was removed with DecontX,^21^ resulting in a high‐quality cell population of 65,958 cells. Harmony algorithm was applied for batch effect correction. Following dimensionality reduction and clustering, annotation and visualization were performed using reference literature^20^ and CellMarker 2.0.^22^ The identification of tumour cells was executed with the inferCNV package, while pseudotime trajectory analysis was conducted using Monocle2.^23^ CytoTrace was employed to assess tissue stemness for validation of differentiation. Additionally, transcriptome data with survival information for liver cancer (TCGA‐LIHC) were acquired from the University of California Santa Cruz Genome Browser, while pan‐cancer datasets, including the Hepatocellular Carcinoma Database (HCCDB),^24^ served as validation sets. After converting the transcriptome data to Transcripts Per Kilobase of exonmodel per Million mapped reads (TPM), we used the limma^25^ package for differential expression analysis. All data necessary for this study are publicly accessible and retrievable from databases.

### 2.2 Single‐cell gene set scoring

Based on single‐cell data, we employed five scoring methods, namely AUCell, UCell,^26^ singscore,^27^ single‐sample gene set enrichment analysis (ssGSEA),^28^ and AddModuleScore,^29^ to evaluate the single‐cell data. Following the normalization of the data, we integrated the datasets to generate a final score. Samples were categorized into 2 groups based on the median score, one characterized by high expression levels and the other by low expression levels, in preparation for the subsequent analysis.

### 2.3 Cellchat analysis

The R package CellChat (version 2.0)^30^ was adopted for cell communication analysis using single‐cell data and our cell categorization. Referring to the CellChatDB.human database, we investigated the interactions occurring among diverse cell types and explored the relationships of 32 different pathways in cellular communication.

### 2.4 SCENIC analysis

The single‐cell regulatory network inference and clustering (SCENIC)^31^ was adopted to delineate unique cellular states within single‐cell datasets and to formulate regulatory networks. Employing pySCENIC, a Python 3.10‐based framework, we scrutinized the regulatory networks of transcription factors (TFs) within malignant tumour subgroups of HCC. The RcisTarget database was leveraged for enrichment analysis to discern regulatory networks linking transcription start sites and genes within HCC. Visualization of these networks was accomplished using the pheatmap package, enhancing an intuitive comprehension of the intricate interactions and regulatory mechanisms operative.

### 2.5 Spatial transcriptomics data processing

We obtained spatial transcriptomic data of primary liver tumours and MLNs from previous studies.^32^ We implemented normalization on the data through the application of SCTransform function, then performed dimensionality reduction with principal component analysis as well as uniform manifold approximation and projection (UMAP), clustering with the default resolution of the first 30 principal components. We visualized gene expression patterns using the SpatialFeaturePlot function. Additionally, we processed spatial transcriptomic data in Python using the Scanpy^33^ package and explored spatial transcriptomic cell interactions with the Stlearn package.

### 2.6 Construction and validation of prognostic models

We created a highly accurate and consistently performing prognostic model by employing a Consensus Integrative Regularized Least Squares strategy. This approach integrated 10 distinct machine learning algorithms along with all 101 of their possible combinations to ensure robustness in predictions. The suite of algorithms included Random Survival Forests (RSF), Elastic Net (Enet), Lasso, Ridge, Stepwise Cox, CoxBoost, Cox Partial Least Squares Regression (plsRcox), Supervised Principal Component (SuperPC), Generalized Boosted Regression Models (GBM), as well as Survival Support Vector Machine (survival‐SVM). The workflow was included: (a) We utilized the FindMarker function to intersect differentially expressed genes (DEGs) between high and low scoring ECs with DEGs of the apelin/APJ gene set to identify key DEGs. (b) These key DEGs were then subjected to 101 algorithmic combinations to fit predictive models. This process was conducted within the framework of Leave‐One‐Out Cross‐Validation (LOOCV), specifically for the TCGA‐LIHC cohort. (c) All models were subsequently tested on the validation set (HCCDB18). (d) For every predictive model, the concordance index (C index), as proposed by Harrell, was determined by examining all the datasets used for validation. The model exhibiting the highest mean C index across these evaluations was recognized as the most effective.

### 2.7 Prognostic model for immune infiltration and immunotherapy prediction

Based on expression data and the ESTIMATE algorithm, the stromal and immune scores were assessed in malignant tumour tissues.^34^ Utilizing the IOBR2^35^ package, the disparities in the distributions of immune cells were computed of high‐risk (HR) and low‐risk (LR) groups using four methods: CIBERSORT,^36^ EPIC,^37^ MCPCounter,^38^ and TIMER.^39^ A Phase II clinical trial,^40^ examined the effectiveness of atezolizumab for bladder cancer, evaluated the model's response to immunotherapy. Additionally, we used the submap algorithm^41^ for the prediction of the degree of response to immunotherapy for the prognostic model and employed the TIP algorithm to predict functional differences in T cells between the two groups.

### 2.8 Cell culture and transfection

Normal vascular endothelial cells (HUVEC) together with the human hepatocellular carcinoma‐derived endothelial cell line (ECDHCC‐1) were obtained from the Typical Culture Reserve Center of China (Chain, Shanghai). The cell cultures were prepared using Dulbecco's modified eagle medium with foetal bovine serum (Gibco, USA), added with penicillin/streptomycin. The cell cultures were maintained under typical laboratory conditions at a temperature of 37°C inside an incubator with a controlled atmosphere comprising 5% carbon dioxide. Transfection was conducted utilzing Lipofectamine 2000 (Invitrogen, USA) to introduce negative control (Vector) and siRNA (Sagon, China) into the cells. The target sequence for the small interfering RNA (siRNA) is as follows:

**Table:** 

Gene	target sequence (5′‐3′)
si apelin1#1	TGCCAAGAATCACAGAATGTTAG
si apelin1#2	CCCACAATAGCCCCAATGTTTGC

### 2.9 Western blot

The process of extracting total proteins was conducted utilizing radio‐immunoprecipitation assay (RIPA, Proteintech, PR20001,) buffer. After cell lysis, the sample was added to a centrifuge tube containing RIPA buffer and subjected to cell disruption using a tube shaker or an ultrasonic homogenizer. The sample was thoroughly mixed to ensure complete cell lysis. The lysate was then centrifuged to remove cell debris and other solid materials. The supernatant, which contains the dissolved membrane proteins, was collected. After the proteins were quantified, samples containing identical amounts of proteins were loaded onto gel electrophoresis using a 10% sodium dodecyl sulphate‐polyacrylamide gel electrophoresis (SDS‐PAGE), followed by their transfer onto a polyvinylidene difluoride (PVDF) membrane with pore sizes of 0.45 μm. The membrane was then treated with a blocking solution consisting of 5% skim milk for a duration of 2 h to prevent nonspecific binding, followed by overnight incubation at a temperature of 4°C with the respective primary antibodies: anti‐ANG2 (24613‐1‐AP, Proteintech, 1/1000), anti‐P‐ERK1/2 (28733‐1‐AP, Proteintech, 1/500), anti‐ERK1/2 (11257‐1‐AP, Proteintech, 1/1000), anti‐KLF2 (23384‐1‐AP, Proteintech, 1/500), anti‐VEGFA (19003‐1‐AP, Proteintech, 1/2000) and anti‐GAPDH (ab8245, 1/5000). Next, the membrane underwent a further incubation with the secondary antibody, which was carried out at ambient temperature for a period of 1 h. Immunoblotting was visualized utilizing an Enhanced Chemiluminescence detection kit (Beyotime, Shanghai). The results were then examined using a Tanon 4600 system (Tanon Science and Technology Co., Ltd.).

### 2.10 Real‐time quantitative reverse transcription polymerase chain reaction

The extraction of total RNA from HUVEC as well as ECDHCC‐1 cell lines was conducted based on TRIzol reagent (Thermo Fisher, USA). cDNA synthesis was performed utilizing HiScript II SuperMix (Vazyme, China) from 500 ng of RNA. PCR amplification was performed under the following conditions: initial denaturation at 94°C for 10 min, followed by cycles of 94°C for 10 s, and 60°C for 45 s. GAPDH was utilized as the internal reference. The primer sequences designed to amplify target genes are provided as follows:

**Table:** 

Gene	Forward primer sequence (5′‐3′)	Reverse primer sequence (5′‐3′)
apelin	CCAGAGGGTCAAGGAATGGGC	ATAACCGCCGGGGGTGGGCA
GAPDH	GTCTCCTCTGACTTCAACAGCG	ACCACCCTGTTGCTGTAGCCAA

### 2.11 Cell viability (CV)

The assessment of CV was conducted utilizing the Cell Counting Kit (CCK)‐8 assay (Beyotime, China). Varying treatments were applied to cells, which were then dispensed at a concentration of 1 × 10^3^ cells per well in a plate with 96 wells. Following the treatments, CCK‐8 solution was added at specified time intervals. After incubation at a temperature of 37°C for 2 h, the absorbance, measured as optical density (OD) at 450 nanometres, for each well was measured via a microplate reader (Thermo Fisher, USA).

### 2.12 Detection of 5‐ethynyl‐2′‐deoxyuridine (EdU)

The proliferation of cells was measured through the application of the BeyoClick™ EdU Cell Proliferation Kit, which incorporated the fluorescent dye Alexa Fluor 594 (Beyotime, Shanghai). Cells were washed with PBS and then cultured with the EdU solution for a duration of 2 h. Subsequently, nuclei were stained with 4′,6‐diamidino‐2‐phenylindole (DAPI) solution. Following additional washing steps, samples were examined through an inverted microscope (Olympus) to visualize EdU incorporation, indicative of cell proliferation.

### 2.13 Angiogenesis Assay

To investigate the influence of apelin on angiogenesis, a tube formation assay was performed utilizing Matrigel Basement Membrane Matrix (Corning, USA, #356234). Initially, each well of a 96‐well plate was filled with the ice‐cold Matrigel (40 μL) and left to solidify at the temperature of 37°C. Next, 1.2 × 10^4^ ECDHCC‐1 cells treated with either si‐NC or si‐apelin were seeded onto the solidified Matrigel. After incubation periods of 3 and 12 h, images of the cells were captured through an inverted optical microscope (Leica, Germany) to evaluate the formation of capillary‐like structures.

## 3 RESULTS

### 3.1 Key Gene Screening and Single‐cell Landscape

**FIGURE 1:**
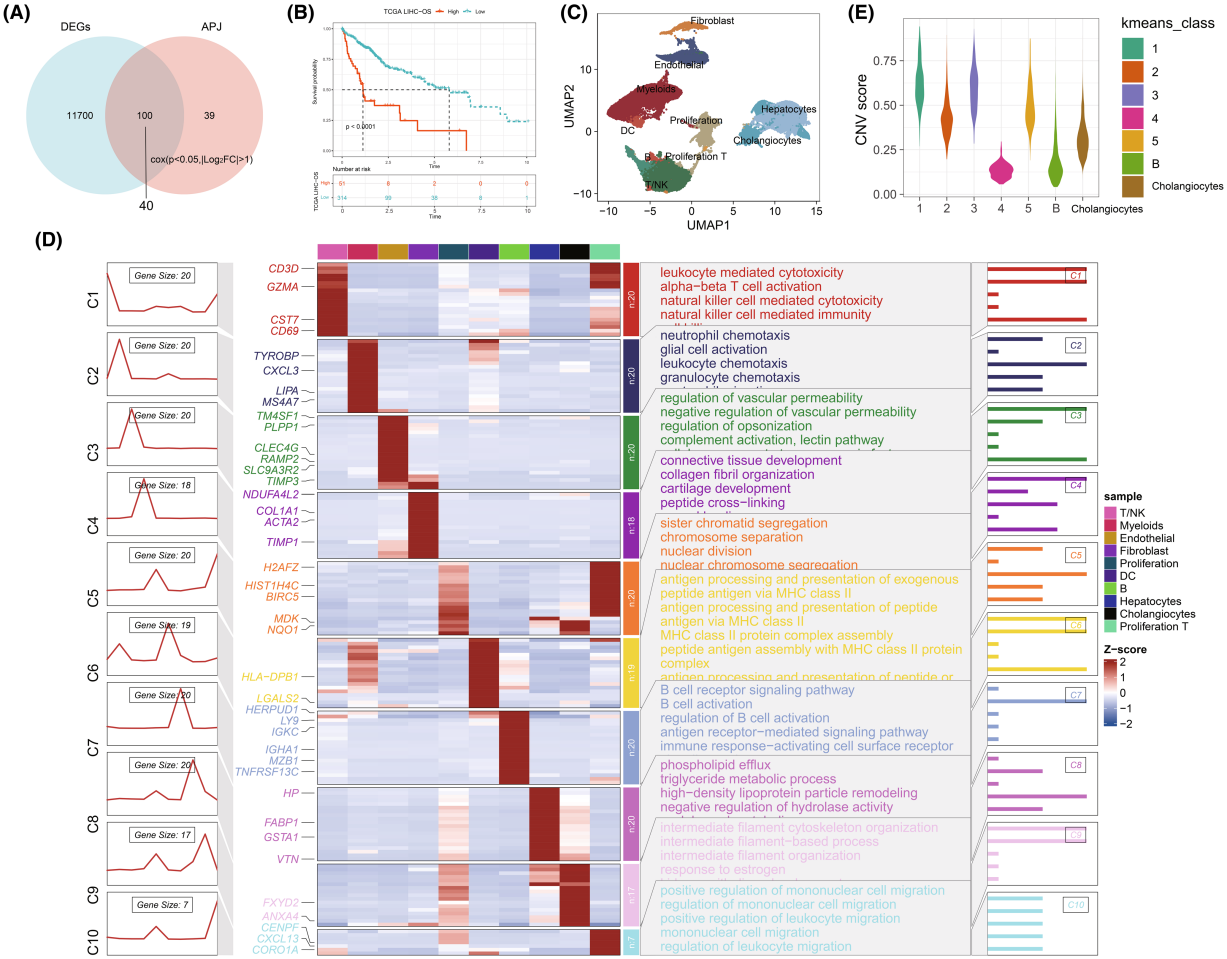
Key gene screening and single‐cell landscape. (A) Venn diagram illustrating the 40 differentially expressed prognostic genes selected through univariate Cox analysis. (B) Kaplan–Meier survival curves demonstrating a worse prognosis in patients with higher expression of these prognostic genes (*p* < 0.001). (C) UMAP plot of the GSE149614 dataset post‐annotation. (D) Heatmap displaying the marker genes for each cell type and their associated functional enrichment. (E) Infercnv‐derived copy number variations for hepatocytes and cholangiocytes to identify malignant cells.

In our study, differential gene expression analysis of tumour and normal tissues was conducted through the R package limma.^25^ The intersection of these DEGs with the apelin/APJ gene set yielded 100 key differential genes. Subsequently, as shown in Figure 1A, univariate Cox analysis identified 40 genes with *p* < 0.05 and |Log2FC| > 1 (APJ‐DEGs). Moreover, Figure 1B shows that patients exhibiting higher expression levels of these DEGs have a poorer prognosis (*p* < 0.05). The GSE149614 dataset was annotated, revealing 10 distinct cell types: T/NK cells, myeloids, ECs, fibroblasts, proliferation cells, proliferation T cells, dendritic cells (DC), B cells, hepatocytes and cholangiocytes (Figure 1C). Functional enrichment analysis corroborated these annotations (Figure 1D). To assess tissue malignancy, infercnv was employed to calculate copy number variants scores (CNVs) for hepatocytes and cholangiocytes. Notably, cluster 4 exhibited low CNV levels, akin to the control group B cells, indicative of normal liver tissue. Conversely, other clusters displayed significant copy number variations. Furthermore, cholangiocytes demonstrated a certain degree of CNVs, consistent with prior research findings (Figure 1E).

### 3.2 Apelin/APJ specifically accumulates in tumour ECs

**FIGURE 2:**
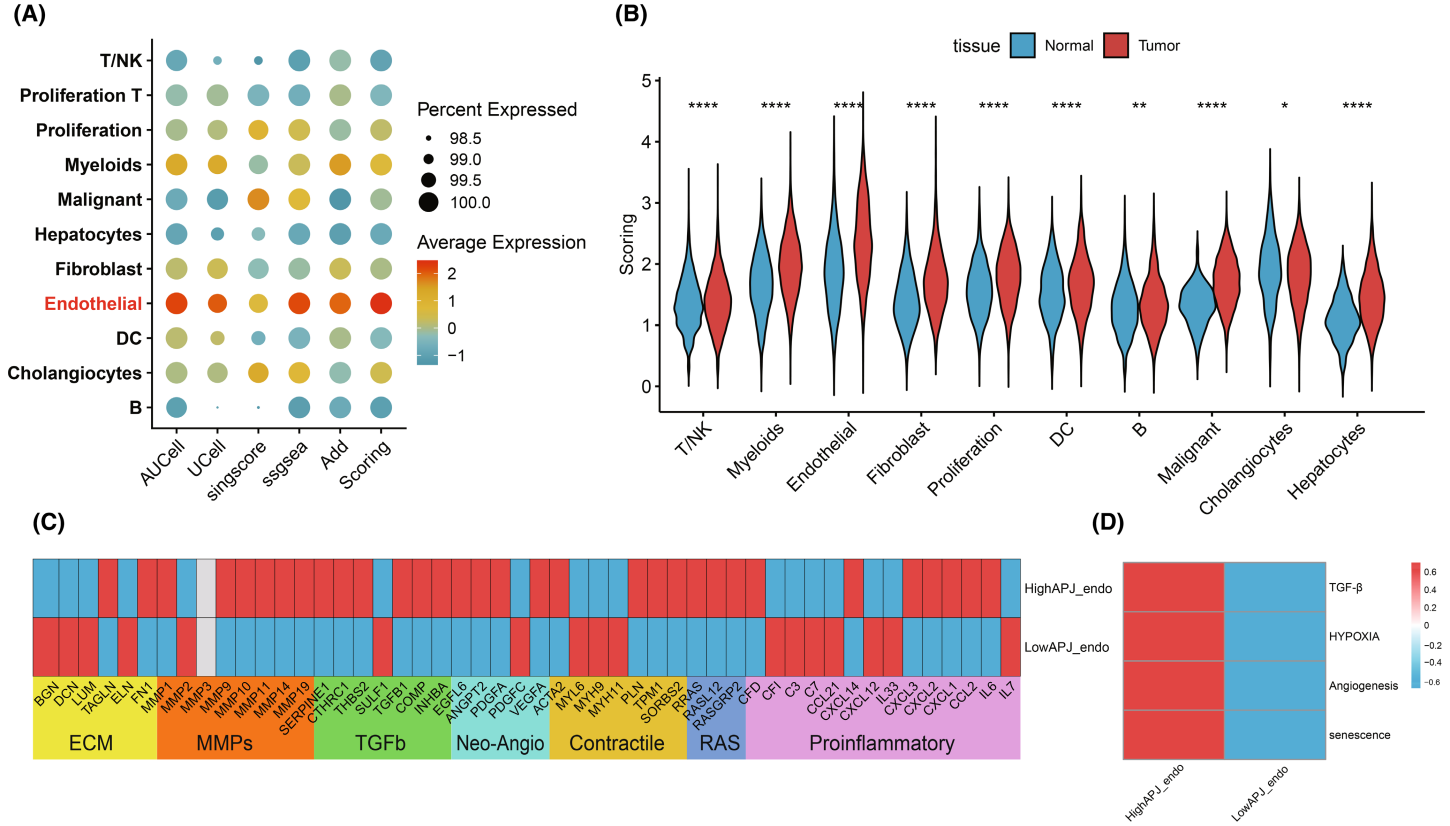
Enrichment analysis of APJ‐DEGs at the single‐cell level in LIHC. (A) Five scoring methods demonstrate specific enrichment of APJ‐DEGs in tumour ECs. (B) Comparative analysis reveals significant enrichment of APJ‐DEGs across all tumour cell types compared to normal samples (*p* < 0.05). (C) Variation in TME gene enrichment between high APJ ECs and low APJ ECs. (D) High APJ ECs demonstrate substantial enrichment of TGF‐β, angiogenesis, hypoxia and senescence pathways, indicating the multifaceted role of APJ in hepatocellular carcinoma beyond angiogenesis promotion. (*p < 0.05, **p < 0.01, ***p < 0.001, ****p < 0.0001).

To elucidate the enrichment of APJ‐DEGs at the single‐cell level in HCC, we applied five different scoring methods to calculate the enrichment score (APJ score). The results revealed a significant enrichment of the APJ score in tumour ECs, which was notably higher than in other cell types (Figure 2A). Interestingly, the APJ score in tumour samples was significantly higher across all cell types compared to normal (*p* < 0.05), with ECs exhibiting the highest degree of enrichment (Figure 2B). However, this difference was not significant in PT, PVTT or MLN. Given these findings and previous research, our attention turned to the differences in APJ scores within ECs. ECs were divided into those with high APJ scores (high APJ ECs0) as well as those with low APJ scores (low APJ ECs) using the median APJ score. Analysis based on TME‐related genes revealed that high APJ ECs significantly enriched in TGF‐β, MMPs, Neo‐Angiogenesis and RAS pathway, whereas low APJ ECs exhibited higher levels of ECM and Proinflammatory enrichment (Figure 2C), suggesting a potentially profound pro‐angiogenic function of high APJ ECs. Subsequent enrichment analysis using TGF‐β and Angiogenesis pathways further supported this hypothesis, showing significant enrichment in high APJ ECs (Figure 2D). Interestingly, in contrast to normal angiogenic factors, Hypoxia and senescence were also significantly enriched, implying that APJ's role in tumours may extend beyond angiogenesis to encompass a multifaceted stimulant.

### 3.3 Subgroup distribution of APJ‐DEGs expression differences across different tumour types

**FIGURE 3:**
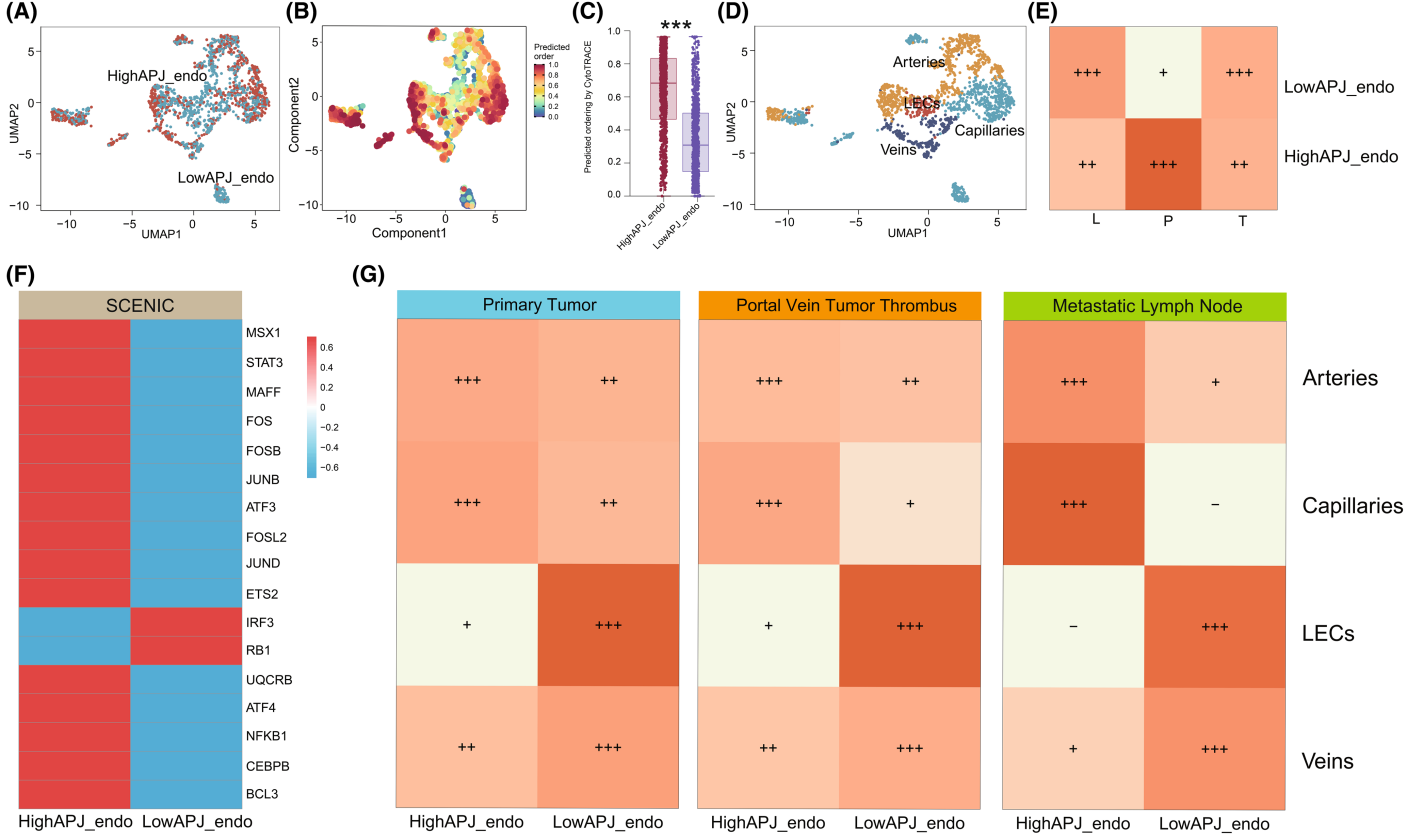
Subgroup distribution of APJ‐DEGs expression differences across different tumour types. (A) Distribution of high and low APJ ECs on a UMAP plot. (B) Differences in stemness between high and low APJ ECs, indicating higher cellular stemness in high APJ ECs. (C) The differential analysis reveals that High APJ endo exhibits significantly higher stemness (*p* < 0.01). (D) UMAP plot showing the subgroup distribution of lymphatic ECs, Arteries, Capillaries and Veins. (E) Subgroup distribution of high and low APJ ECs in PT, PVTT and MLN. (F) SCENIC analysis demonstrating higher transcriptional activity in high APJ ECs. (G) Subgroup distribution of high and low APJ ECs across lymphatic ECs, arteries, capillaries and veins. EC, endothelial cell; PT, primary tumour; PVTT, portal vein tumour thrombus; MLN, metastatic lymph nodes.

Upon extraction of ECs, we re‐dimensionally reduced and re‐clustered them. Figure 3A illustrates the distribution of high APJ ECs and low APJ ECs on a UMAP plot. Using Cytotrace, we calculated the stemness of high APJ ECs and low APJ ECs, revealing that high APJ ECs possesses higher stemness than low APJ ECs (Figure 3B). Cytotrace scoring, upon quantification, further confirmed that high APJ expression is associated with increased stemness (*p* < 0.01) (Figure 3C). Following literature reference,^42^ we further classified ECs into Lymphatic ECs, Arteries, Capillaries and Veins (Figure 3D). Interestingly, we observed a richer subgroup distribution of high APJ ECs in PVTT (Figure 3E), suggesting that the apelin/APJ axis might play a role in promoting tumour hematogenous metastasis. Scenic analysis also revealed markedly rich transcriptional activity in high APJ ECs compared to low APJ ECs, including TFs associated with tumour progression and pro‐angiogenic pathways such as NFKb, JUNK‐STAT, CEBPB (Figure 3F). Subsequent subgroup distribution indicated a more significant enrichment of Capillaries and Arteries in high APJ ECs, and within PVTT, the enrichment of Capillaries was even more pronounced (Figure 3G). Additionally, we observed almost no enrichment of LECs in high APJ ECs, suggesting a potential link to immune suppression within the tumour.

### 3.4 Pseudotime and Cellchat analyses

**FIGURE 4:**
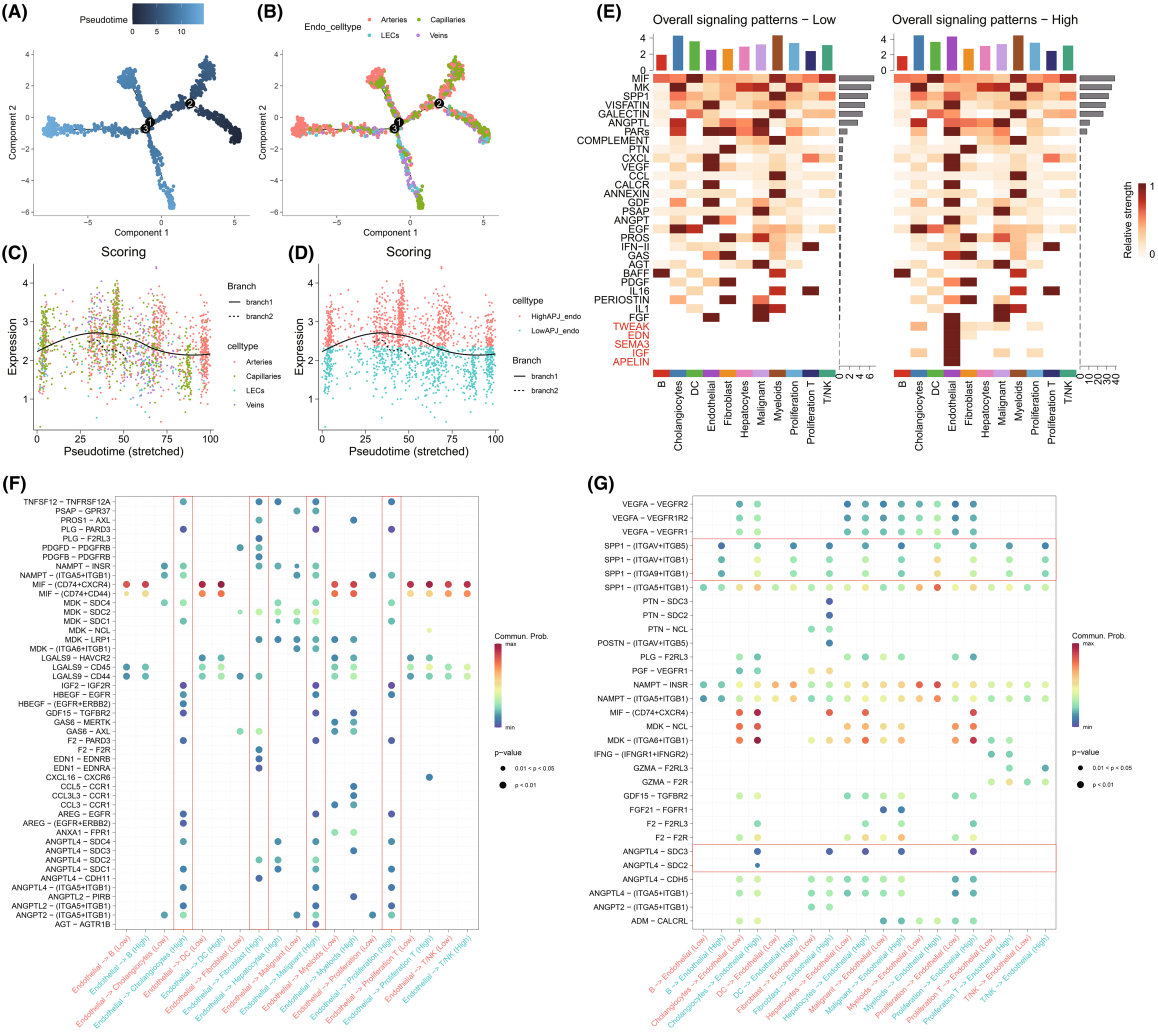
Pseudotime and cell communication analyses. (A) Pseudotime analysis of ECs. (B) Differentiation trajectories of Lymphatic ECs, Arteries, Capillaries and Veins revealed by Pseudotime analysis. (C, D) Expression trends of APJ score during EC differentiation process. (E) Heatmap showing differential cell communication heatmap between high and low APJ ECs. (F) Bubble Chart depicting ECs as ligands in cell communication. (G) Bubble chart illustrating ECs as receptors in cell communication. EC, endothelial cell.

Monocle2 analysis revealed that during the development of vascular endothelium, Capillaries are positioned at the developmental starting point and extend throughout the endothelial differentiation trajectory, while Arteries and Veins are situated toward the end of development (Figure 4A,B). This suggests that Capillaries have higher stemness, aligning with previous conclusions and indicating that the apelin/APJ system may promote tumour metastasis by facilitating the formation of intratumoural microcapillaries. Furthermore, scoring based on APJ‐DEGs showed that the APJ score gradually increases at the beginning and middle stages of development and decreases as differentiation matures (Figure 4C,D). Subsequent differential cell communication analysis of high / low APJ ECs and other cell types indicated that pathways such as apelin, insulin‐like growth factor (IGF), SEMA3, END and TWEAK are exclusively present in high APJ ECs (Figure 4E). When ECs act as ligands, high APJ ECs engage in cell communication with malignant, cholangiocytes, proliferation, etc., involving pathways related to ligands such as angiopoietins (ANGPLs), heparin‐binding EGF‐like growth factor (HBEGF), and IGF; and with fibroblasts, they exhibit cell communication related to EDNs and PDGFs pathways. Additionally, myeloid cell communication is upregulated with CCL3L3‐CCR1, CCL‐CCR1 ligands (Figure 4F). When ECs act as receptors, cell communication between immune cells and high APJ ECs is significantly upregulated in the SPP1‐ITGs and ANGPTL4‐SDC3 ligand‐receptor pathways (Figure 4G).

To clarify the regulatory impacts of differential ligands on downstream genes, we employed Nichenet for additional cell communication analysis. The results revealed that ligands such as transforming growth factor beta 1 (TGFB1) as well as bone morphogenetic proteins (BMPs) play significant roles in cell communication (Figure S1A). Additionally, the differential Nichenet analysis highlighted that the TGFB1, BMPs and VEGF signalling pathways are the primary cell communication pathways that differ between high and low APJ ECs (Figure S1B). This analysis underscores the crucial roles these signalling pathways play in modulating cellular interactions and functions in different endothelial subtypes, potentially influencing tumour progression and metastasis dynamics.

### 3.5 High and low APJ ECs, along with spatial pseudotime and spatial cell communication

**FIGURE 5:**
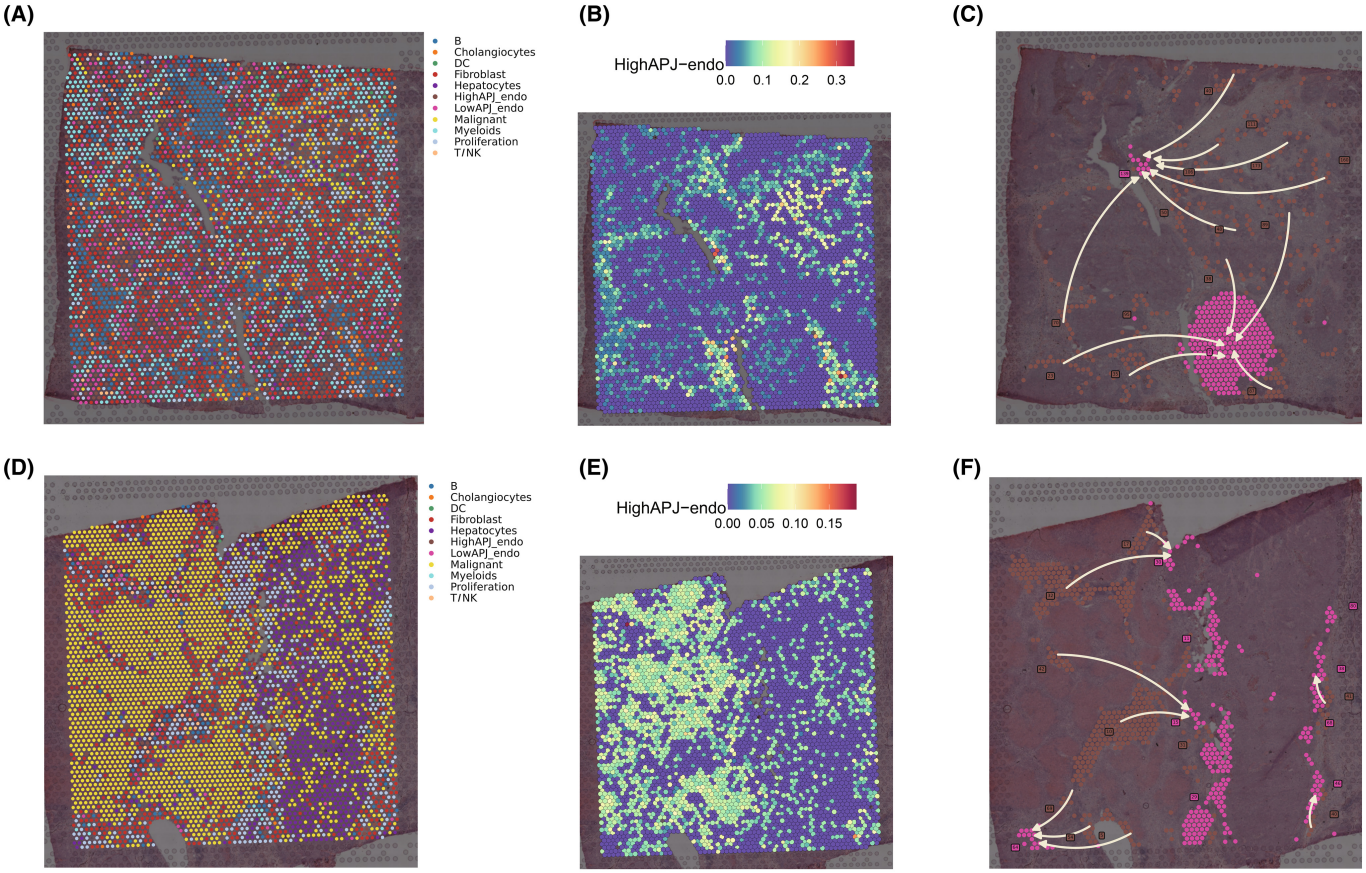
Spatial distribution and Pseudotime analysis of high and low APJ ECs. (A) Deconvolution for spatial localization in the tumour section. (B) Heatmap illustrating the spatial enrichment of high APJ ECs in the tumour section. (C) Pseudotime differentiation trajectory from high APJ ECs to low APJ ECs in the tumour section. (D) Deconvolution for spatial localization in the leading‐edge section. (E) Heatmap depicting the spatial enrichment of high APJ ECs in the leading‐edge section. (F) Pseudotime differentiation trajectory from high APJ ECs to low APJ ECs in the leading‐edge section.

Utilizing single‐cell RNA sequencing data, we conducted deconvolution to spatially localize different cellular components within the main tumour mass and its leading‐edge sections. The results indicate that high APJ ECs are predominantly situated within the tumour nests and in the adjacent periphery, while low APJ ECs exhibit a more dispersed distribution (Figure 5A,B). Furthermore, the leading‐edge section exhibits a more pronounced infiltration of malignant tissue, with high APJ ECs being significantly enriched around the tumour nests (Figure 5D,E), suggesting that high APJ ECs might be crucial in facilitating the spread of cancerous tissue into adjacent healthy tissues. Pseudotime analysis also confirms the trajectory of high APJ ECs differentiating from the periphery of the tumour nests towards the external low APJ ECs, in line with previous findings (Figure 5C, D). Results of cell communication indicate that at the spatial level, high APJ ECs exhibit a high level of cell communication with myeloid cells, malignant cells and fibroblasts (Figure S2A), whereas low APJ ECs demonstrate a lower correlation with other cells (Figure S2B).

### 3.6 Prognostic model construction

**FIGURE 6:**
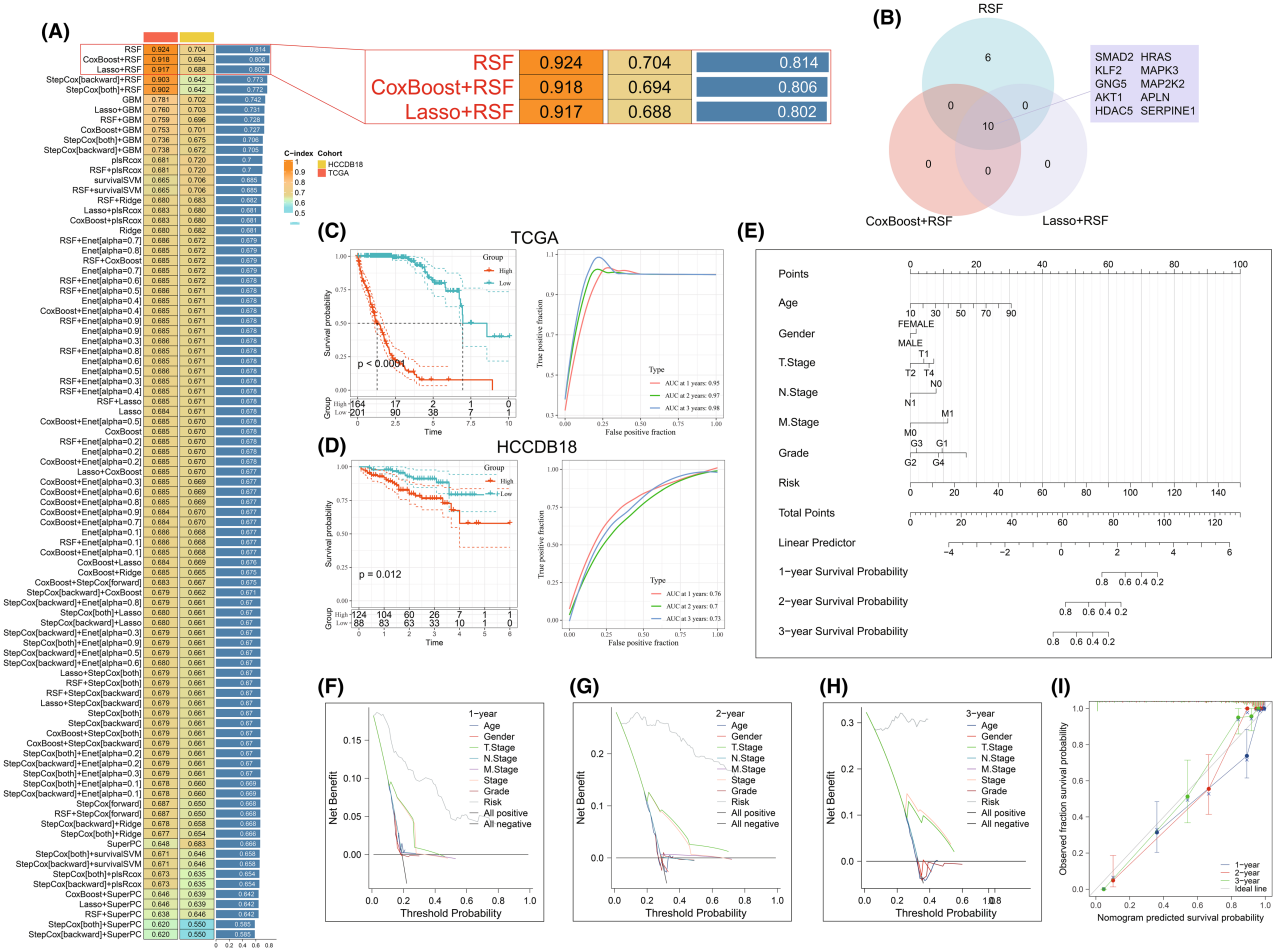
Prognostic model construction. (A) Construction of the prognostic model. (B) Selection of the top three models, with CoxBoost + RSF chosen as the prognostic model. (C) Prognostic curves and AUC for the TCGA‐LIHC model. (D) Prognostic curves and AUC for the HCCDB18 model. (E) Nomogram for predicting prognosis. (F–H) Decision curve analysis for 1, 2 and 3 years, respectively. (I) Calibration curves for 1, 2 and 3 years, respectively.

We utilized the Findmarker function to identify DEGs between high and low APJ ECs. Gene Ontology (GO) analysis showed that these DEGs were predominantly enriched in processes associated with cell adhesion as well as extracellular matrix (ECM) construction. KEGG pathway analysis highlighted that primary differences in pathways were concentrated on cellular senescence, apelin signalling pathway, along with PI3K‐Akt signalling pathway (Figure S3A). In addition, GSEA showed that the functional differences between the two EC subpopulations were concentrated in pathways intimately linked to tumour inflammation alongside immune regulation, including cell adhesion, NF‐κB signalling pathway, IL‐2‐STAT5 signalling pathway, hypoxia and apoptosis (Figure S3B). Subsequently, we intersected the DEGs with APJ‐DEGs to obtain a set of 16 key DEGs. Univariate Cox analysis confirmed that all these genes were prognostic. Utilizing these 16 key genes, a prognostic model was established using an integrated machine learning approach. Within the TCGA‐LIHC cohort, 101 predictive models were fitted using a LOOCV framework; Meanwhile, the c‐index scores were computed for every model using the validation set (HCCDB18) (Figure 6A). We compared the c‐index scores of the top three models, which were quite similar, and found that the CoxBoost + RSF and lasso + RSF models both included 10 genes (Figure 6B). Therefore, we selected the CoxBoost + RSF model as our prognostic model. The TCGA‐LIHC and HCCDB18 models demonstrated excellent performance, with the HR group showing poorer prognosis (*p* < 0.05). Besides, the area under the curve (AUC) for the training set at 1‐year, 2‐year and 3‐year period was 0.95, 0.97 and 0.98, respectively, and for the HCCDB18 validation set, it was 0.76, 0.7 and 0.73. These values suggest that the prognostic model has a strong predictive performance (Figure 6C,D). Next, a nomogram was constructed utilizing the established model in conjunction with data, which showed high predictive accuracy (*p* < 0.01) (Figure 6E). Decision curve analysis (DCA) indicated that the prognostic model offered better clinical utility compared to other indicators at 1, 2 and 3 years (Figure 6F–H). Meanwhile, the calibration curve also revealed high accuracy of the model (Figure 6I). Additionally, integrating clinical information indicated that the HR group was in more progressed stages of the disease and faced a more unfavourable prognosis (*p* < 0.01) (Figure S4A), and the HR group in the validation set had a higher frequency of Portal Vein Invasion (*p* < 0.001) (Figure S4B), which aligned with our previous hypotheses.

### 3.7 Immune infiltration and prediction of the response to immunotherapy

**FIGURE 7:**
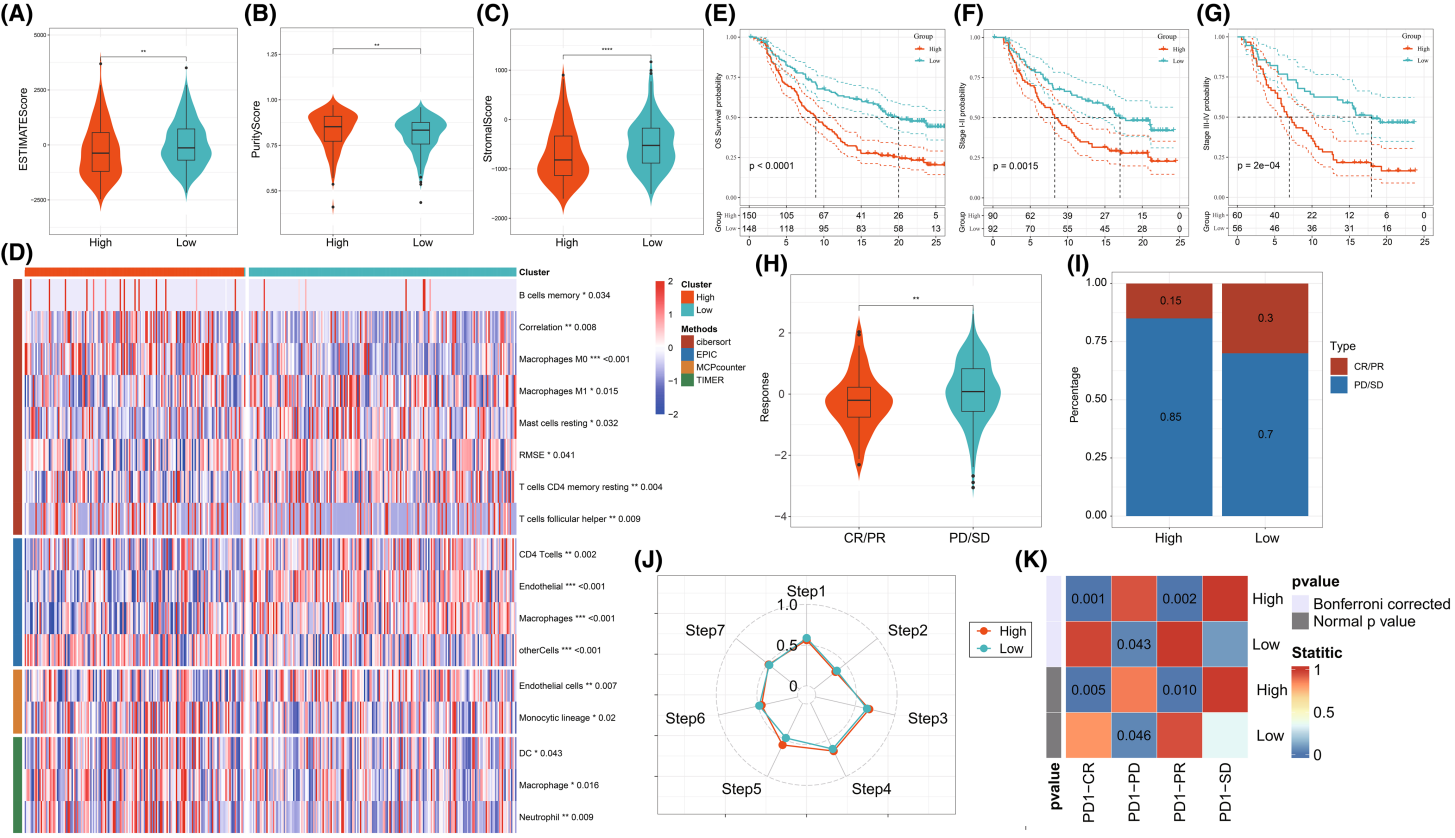
Immune infiltration and prediction of the response to immunotherapy. (A) Estimate score of the HR and LR groups. (B) Stromal score of the HR and LR groups. (C) Purity score of the HR and LR groups. (D) Immune infiltration among the HR and LR groups assessed by four methods. (E) Worse prognosis of the HR group among the IMvigor210 cohort (*p* < 0.001). (F) Worse prognosis of the HR group among stage I‐II individuals among the IMvigor210 cohort (*p* < 0.001). (G) Worse prognosis of the HR group among stage III‐IV individuals among the IMvigor210 cohort (*p* < 0.001). (H) Poorer response to atezolizumab immunotherapy among the HR group. (I) Higher proportion of PD/SD in the HR group. (J) Differences in anti‐tumour immune activity between the HR and LR groups evaluated by the TIP algorithm. (K) Sensitivity to PD‐1 immunotherapy subtype predicted by the Submap algorithm for the HR group. *p < 0.05, **p < 0.01, ***p < 0.001.

The immune scores for both the HR and LR groups were determined using the Estimate package. Results showed significantly lower estimate score and stromal score among the HR in contrast with the LR group (*p* < 0.05). Meanwhile, a higher purity score was observed among the HR group (*p* < 0.01) (Figure 7A–C). This suggests that higher tumour purity and less stromal content in the HR group may favour tumour proliferation and metastasis. Next, the variations within immune subpopulations between the high and LR groups were assessed using four different immune infiltration algorithms. Results showed that in contrast with the LR group, the T cell subpopulations within the HR group were reduced with statistical significance, suggesting a possible state of immune suppression (Figure 7D). The IMvigor210 cohort was then utilized to demonstrate the model's ability concerning the efficacy of immunotherapy treatments. We found that the model was also applicable to other cancers, with significant prognostic differences observed across all clinical stages within the IMvigor210 cohort, with the HR group exhibiting poorer outcome (Figure 7E–G). Additionally, the HR group demonstrated a less favourable response to the treatment with atezolizumab (*p* < 0.05) (Figure 7H), with a higher proportion of patients experiencing progressive disease (PD) or stable disease (SD) (Figure 7I). The tumour inflammation signature (TIP) algorithm assessment of antitumour immune activity indicated significantly lower activity in cancer‐immunity cycle including release of cancer cell antigens (Step 1), cancer antigen presentation (Step 2), recognition of cancer cells by T cells (Step 6) in the HR group in contrast with the LR group. However, in infiltration of immune cells into tumours (Step 5), the HR group exhibited significantly higher activity than the LR group (Figure 7J). The submap algorithm evaluation that there was a potential for the HR group to respond positively to PD‐1 immunotherapy (Figure 7K). As shown in Figure S5A–F, drug prediction analysis further revealed a less favourable response to medications such as axitinib, bicalutamide, dasatinib, erlotinib, gefitinib and lapatinib among the HR group (*p* < 0.05).

### 3.8 Hub genes and their relationships with immune and pathway interactions

**FIGURE 8:**
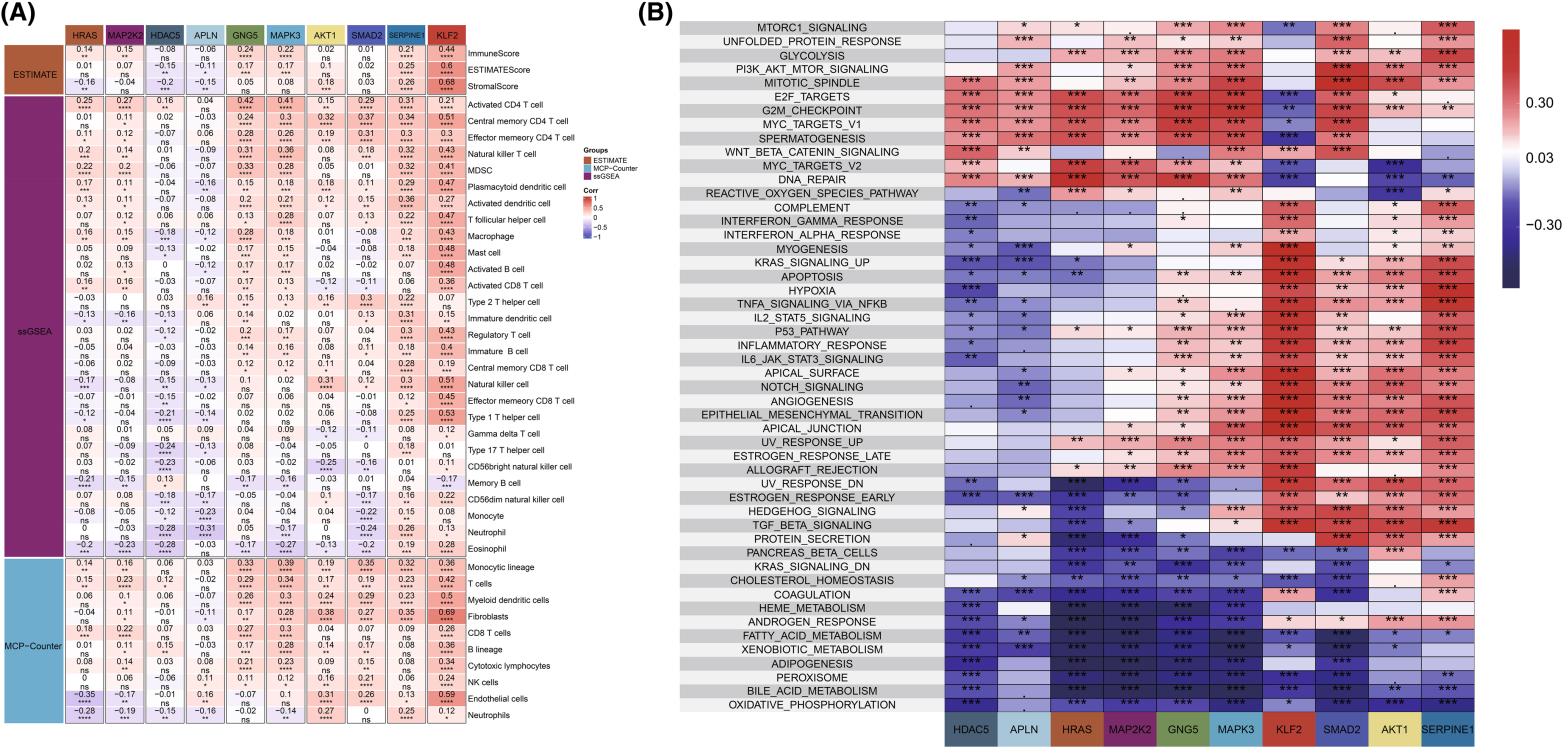
Relationships of key genes with the immunity and the associated pathways. (A) Correlation of key genes with immune cells. (B) Pathway enrichment of key genes. *p < 0.05, **p < 0.01, ***p < 0.001, ****p < 0.0001.

We utilized the methods of ESTIMATE^34^ together with MCP‐counter^38^ to assess the immune scores of the TCGA‐LIHC dataset. Additionally, we extracted genes for 28 immune cell types from a referenced literature^43^ and then evaluated the scores for each sample using the ssGSEA method. Apelin and HDAC5 were found to be negatively linked to most immune cell subtypes (Figure 8A), suggesting that in the HR model, apelin and HDAC5 may be associated with an immunosuppressive environment under high APJ score conditions. Furthermore, apelin and others were positively correlated with pathways like the PI3K‐AKT signalling pathway as well as the WNT signalling pathway, which were linked to tumour progression and proliferation, while KLF2, SAMD2, AKT1 and SERPINE1 were closely related to the NF‐κB signalling pathway, the IL‐2‐STAT5 signalling pathway, and the Hypoxia tumour validation pathway (Figure 8B). Finally, we visualized the functional roles and pathway positions of the model genes in the apelin/APJ pathway, revealing that apelin is the initiating gene of the apelin/APJ signalling pathway. The model genes were mainly enriched in the apelin‐HDAC5‐KLF2 angiogenesis axis and mediated the occurrence of the PI3K‐AKT signalling pathway as well as the MAPK signalling pathway, providing direction for targeting the apelin/APJ pathway in liver cancer (Figure S6).

### 3.9 Pan‐cancer analysis of a single gene

**FIGURE 9:**
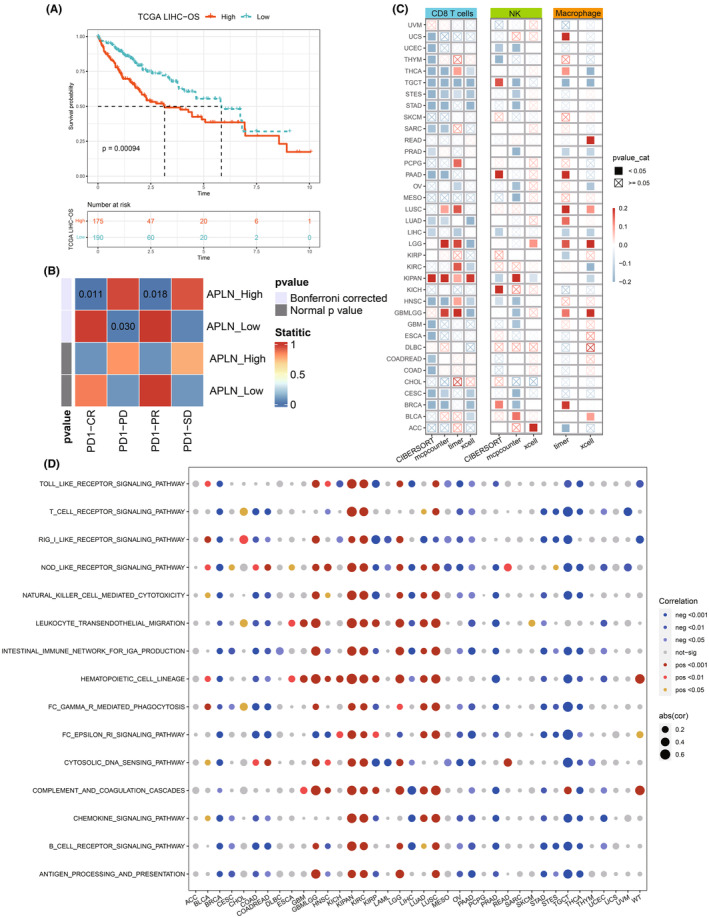
Pan‐cancer analysis of a single gene. (A) LIHC patients with high apelin expression demonstrate poorer prognosis (*p* < 0.001). (B) Apelin expression predicts response to PD‐1 therapy. (C) Pan‐cancer analysis involving the correlation of apelin expression with CD8 T cells, NK cells, alongside macrophages across different cancer types. (D) Pan‐cancer GSEA analysis illustrating the association of high / low apelin expression with immune response‐related pathways.

We identified apelin as one of the most significant genes in our model, and thus conducted a single‐gene analysis on apelin. Results indicated the poor prognosis in LIHC individuals with high apelin expression (*p* < 0.001) (Figure 9A). Additionally, Submap analysis suggested that patients with high apelin expression might benefit from immunotherapy (Figure 9B). Pan‐cancer immune infiltration analysis revealed that apelin was in a negative correlation with CD8 T cells, NK cells, as well as macrophages within most tumours, except for lung squamous cell carcinoma (LUSC), low‐grade glioma (LGG), as well as pan‐kidney cohort (KIPAN) (Figure 9C), suggesting a potential role in immunosuppression within the TME. Interestingly, a pan‐cancer single‐gene GSEA analysis using immune‐related pathways in KIPAN, kidney renal clear cell carcinoma (KIRC), as well as LUSC, potentially acting as an immune‐promoting gene, whereas in other tumours, it often functioned as an immune‐suppressive gene (Figure 9D), highlighting the heterogeneity of tumours.

### 3.10 Apelin promoted the activity of ECDHCC‐1 and its angiogenic capabilities

**FIGURE 10:**
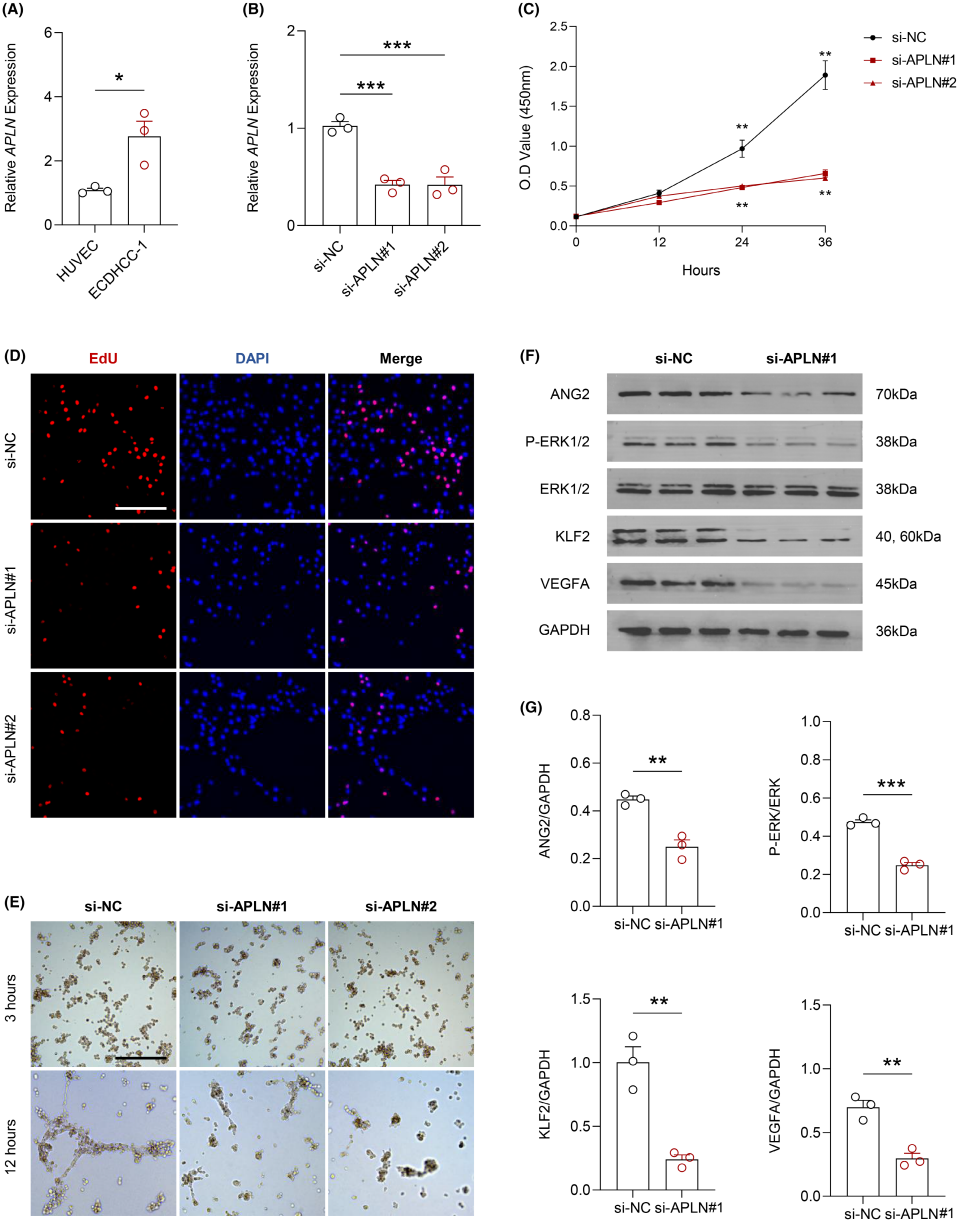
Effect of APLN on the modulation of the viability and angiogenic potential of ECDHCC‐1 cells. (A) Transcriptional levels of APLN in HUVEC and ECDHCC‐1 cell lines, with relative quantification analysis; (B) Validation of the inhibitory efficiency of small interfering RNA targeting APLN transcription in ECDHCC‐1 cells using PCR, accompanied by relative quantification analysis; (C) CCK8 assay detecting alterations in cell viability in ECDHCC‐1 cells following APLN expression interference; (D) EdU staining assessing changes in cell proliferation ability in ECDHCC‐1 cells post APLN expression interference; (E) *In vitro* tube formation assay monitoring changes in angiogenic capability of ECDHCC‐1 cells before and after APLN expression interference; (F, G). Western blot analysis of alterations in downstream angiogenesis‐related molecules in ECDHCC‐1 cells post interference with APLN expression.

We initially assessed the transcription levels of APLN in HUVEC and ECDHCC‐1, with PCR results indicating a significant upregulation of APLN transcription in ECDHCC‐1 (Figure 10A). To examine the impact of apelin on the viability and angiogenic capability of ECDHCC‐1 cells, we first constructed two sets of small interfering RNAs (si‐APLN#1 and si‐APLN#2) and evaluated their effect on APLN expression in ECDHCC‐1, with PCR results showing that both siRNAs could significantly inhibit apelin transcription in ECDHCC‐1 (Figure 10B). Subsequently, we investigated the activity and proliferation of ECDHCC‐1 following apelin knockdown using CCK8 assays and EdU staining (Figure 10C,D). The results explicitly indicated that APLN knockdown significantly inhibited both the activity and proliferation capabilities of ECDHCC‐1 cells. Given that ECDHCC‐1 is a cancer cell line originating from vascular endothelium with potent angiogenic capabilities, in vitro tube formation assays demonstrated that inhibiting APLN expression significantly reduced the angiogenic capacity of ECDHCC‐1 in vitro (Figure 10E). To elucidate the mechanism by which APLN promotes angiogenesis, we examined the expression of downstream molecules related to angiogenesis before and after APLN knockdown in ECDHCC‐1. Based on the comprehensive results of CCK‐8 and EdU assays, we concluded that si‐APLN#1 exhibited superior efficacy. Therefore, subsequent Western blot experiments were conducted using si‐APLN#1. WB results suggested that after APLN knockdown, the levels of ANG2, KLF2, VEGFA and the phosphorylation level of ERK1/2 significantly decreased within ECDHCC‐1 cells (Figure 10F,G).

## 4 DISCUSSION

Numerous studies have demonstrated that the Apelin/APJ axis is significantly overexpressed in various cancers, including lung adenocarcinoma,^14^ breast cancer,^44^ and HCC.^45^ Additionally, tumour cells with high levels of Apelin/APJ expression exhibit an increased tendency for metastasis. Research indicates that Apelin can enhance the expression of MMP‐2 through the activation of the PI3K/Akt pathway and FOXO3A, leading to the degradation of the ECM and promoting the motility and migration of vascular smooth muscle cells (VSMCs), which facilitates tumour invasion and metastasis.^46^ Hall et al.^15^ demonstrated that treating human lung cancer cell line H1299 with the APJ antagonist ML221 resulted in a reduction in the levels of migratory markers such as Vimentin, MMP‐9 and MMP‐3, subsequently inhibiting tumour invasion. Similar outcomes were observed in colon cancer cell lines,^18^ potentially attributed to the upregulation of filamentous actin and cofilin, which were inhibited following ML221 treatment. Moreover, angiogenesis induced by the Apelin/APJ axis is another critical factor contributing to tumour progression. Evidence shows that both in vivo and in vitro overexpression of APLN increases Sirt3 levels, upregulating VEGF/VEGFR‐2 and Ang‐1/Tie‐2, thus enhancing vascular density.^18^ In human non‐small cell lung cancer (NSCLC) specimens, APLN expression correlates positively with capillary size and microvessel density (MVD). Tumour samples exhibiting high APLN expression also show increased capillary dimensions and microvessel quantities. Additionally, subcutaneous injection of APLN‐transfected NSCLC cells in nude mice significantly elevated MVD within tumours.^14^ Treatment of HCC model mice with the APJ antagonist F13A resulted in a notable decrease in MVD and arterial vessel density (AVD). Furthermore, MVD and AVD are found to be higher in tumour tissues compared to non‐tumour tissues, with a greater number of APJ‐positive vessels identified within tumour regions. The elevation of MVD and AVD in tumour tissue is likely due to the overexpression of APJ in tumour‐associated vasculature and the activation of the APJ signalling pathway by Apelin.^47^ These findings underscore the critical role of the Apelin/APJ axis in tumour biology.

To the best of our knowledge, this represents the initial study to comprehensively investigate into the function of apelin/APJ in HCC from a multi‐omics perspective. Apelin/APJ was significantly overexpressed in the vascular endothelium of HCC, whereas its expression was lower in normal samples, in line with the findings of Zhou et al.^48^ Interestingly, vascular ECs with high APJ expression exhibited increased stemness and were significantly enriched in PVTT. Previous studies have shown that apelin/APJ promoted tumour vascular remodelling,^49^, ^50^ thereby increasing the likelihood of hematogenous metastasis. Additionally, secondary annotation of vascular ECs indicated that high APJ ECs was more enriched in capillaries, leading us to hypothesize that apelin/APJ might promote the growth and advancement of tumours by facilitating the generation of capillaries within the tumour. Previous research^16^ demonstrated that apelin knockout increases NK cell production and inhibited myeloid‐derived suppressor cells (MDSCs) in a mouse model of breast cancer. Moreover, apelin downregulated the expression of macrophage‐associated inflammatory proteins to suppress immune‐related inflammatory responses.^51^ We found that the expression of liver ECs in high APJ ECs was minimal, which may be one of the key factors through which apelin/APJ influences the TME.

Wang et al.^52^ discovered that apelin promoted the proliferation as well as the metastasis of cervical cancer cells through the PI3K/AKT/mTOR pathway. Our analysis of cell communication also indicates significant expression of pro‐angiogenic pathways involving ANGPLs, HBEGF, IGF and others in high APJ ECs. Moreover, pathways related to TGF‐β, oxidative stress and PI3K‐AKT are notably active in high APJ ECs. Spatial transcriptomics analysis further reveals the predominant clustering of high APJ ECs around tumour nests, providing additional evidence that the apelin/APJ axis may contribute to tumour growth and metastasis by remodelling tumour‐associated vasculature, thereby supporting our previous findings.

To further investigate and validate the impact of the apelin/APJ pathway on HCC and its potential mechanism in promoting tumour metastasis through PVTT formation, a prognostic model was developed using key genes associated with apelin/APJ using machine learning techniques. The model demonstrated excellent predictive performance, as evidenced by the AUC, calibration curves, as well as DCA. Moreover, analysis of patients' clinical data revealed that the HR group were more susceptible to portal vein invasion, suggesting a potential role of apelin/APJ in promoting tumour metastasis through the formation of PVTT. Subsequent analysis of immune infiltration demonstrated significant immune suppression in the HR group, implying the possible challenges in chemotherapy and immunotherapy responses. Further investigation into the genes contributing most to the model identified apelin as a key upstream gene in the apelin/APJ pathway. Apelin was found to promote angiogenesis and mediate the upregulation of the PI3K‐AKT signalling pathway through the apelin‐HDAC5‐KLF2 axis, providing a potential avenue for future research. Pan‐cancer analysis of single genes also suggested that apelin may inhibit immune effector cells across various tumour types, highlighting its importance as a potential target for tumour angiogenesis.

Certainly, our research had certain limitations that warrant acknowledgment. Firstly, despite demonstrating the prognostic efficacy of the apelin/APJ‐based prognostic model using data from TCGA and GEO databases, further validation with large cohorts of clinical samples is imperative to ensure its robustness and generalizability. Secondly, deeper exploration of the mechanisms underlying apelin‐mediated angiogenesis in HCC is warranted. This area will be the primary focus of our subsequent research endeavours.

## 5 CONCLUSION

In summary, we developed a prognostic model utilizing apelin/APJ pathways, integrating bulk transcriptome, spatial transcriptome and single‐cell data analyses. Based on a multi‐omics approach, we shed light on the role of apelin/APJ in HCC. Through experimental validation of key genes, we provided new insights into potential targets for anti‐angiogenic therapy in HCC.

## AUTHOR CONTRIBUTIONS

**Yongfu Zhu:** Conceptualization (equal); data curation (equal); resources (equal); writing – original draft (equal). **Pengcheng Zhang:** Conceptualization (equal); formal analysis (equal); resources (equal); writing – original draft (equal). **Xingxing Huo:** Data curation (equal); investigation (equal); visualization (equal). **Yi Ling:** Methodology (equal); validation (equal). **Xiang Lv:** Formal analysis (equal); funding acquisition (equal); investigation (equal); project administration (equal); writing – review and editing (equal). **Shengyou Lin:** Investigation (equal); project administration (equal); writing – review and editing (equal). **Hang Song:** Project administration (equal); writing – original draft (equal); writing – review and editing (equal).

## FUNDING INFORMATION

This article was supported by the 2023 Anhui Provincial Natural Science Foundation (NO. 2308085MH296), as well as the 2022 Research Projects of Anhui Provincial Health Commission (NO. AHWJ2022b053), and the Bozhou Vocational and Technical College Special Research Project on Subsidy for Enhancing Medical Service and Assurance Capacity and 2022 University Scientific Research Project of Anhui University of Chinese Medicine (NO. 2022AH050415) and Shanghai Municipal Health Commission General Project (NO. 202340139), and Key Project of Anhui Provincial Department of Education (grant number 2023AH050870).

## CONFLICT OF INTEREST STATEMENT

The authors declare no competing interests.

## Supporting information


Appendix S1.

## Data Availability

The datasets used and analysed during the current study are available from the corresponding author on reasonable request.
