# Supplementary material for: Single‐cell and spatial transcriptomics reveal apelin/APJ pathway's role in microvessel formation and tumour progression in hepatocellular carcinoma
Source: J Cell Mol Med. 2024 Oct 21;28(20):e70152. doi: 10.1111/jcmm.70152 (PMC11493554; doi:10.1111/jcmm.70152)
Supplement: Supplementary file 1 — Appendix S1. [file JCMM-28-e70152-s001.docx]

**Supplementary Figure.**

**
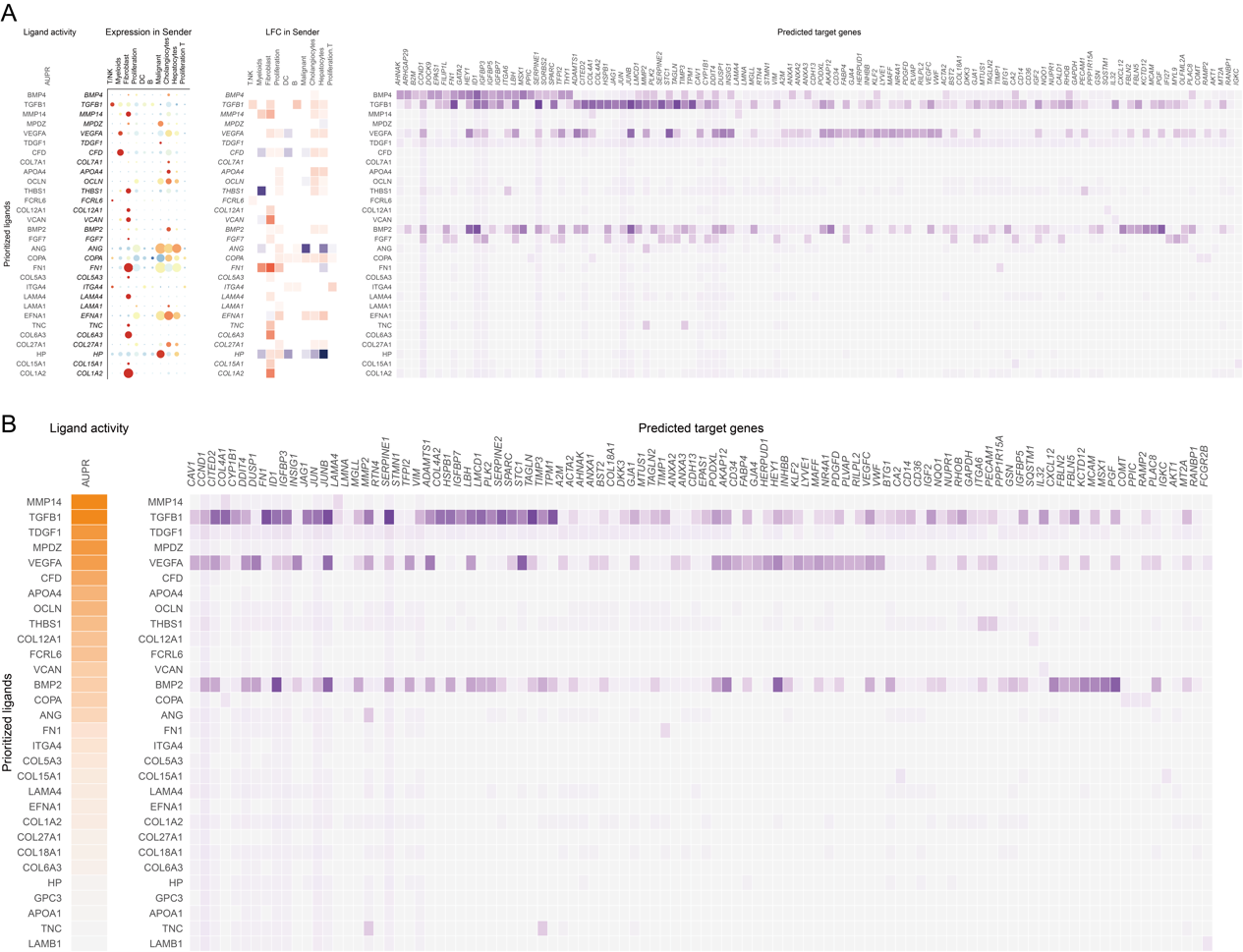
**

**Supplementary Figure.1** Nichenet and Cellchat analyses. **(A)**. Visualization of the expression of top-predicted ligands and their target genes in a combined heatmap. **(B)**. Differential ligands and their target genes between high and low APJ expression.


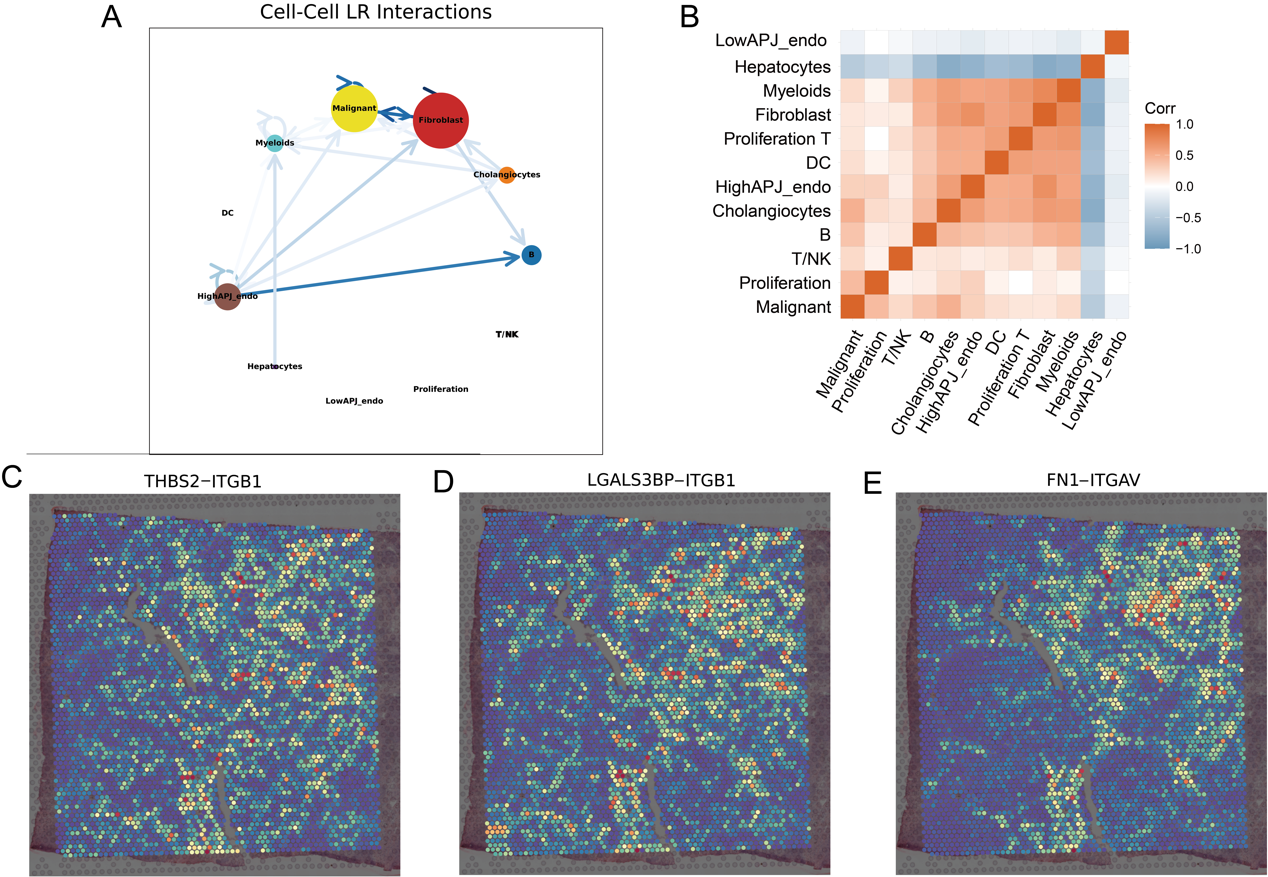


**Supplementary Figure.2** Spatial cell communication. **(A)**. The spatial cell communication network diagram illustrates that high APJ ECs exhibit a higher intensity of cell communication with other cells. **(B)**. The spatial cell correlation heatmap indicates that low APJ ECs have a lower correlation with other cells. **(C-E)**. Heatmaps depicting the distribution of the top 3 cell communication ligands show that the ligand pairs are enriched and clustered in the tumor nests.


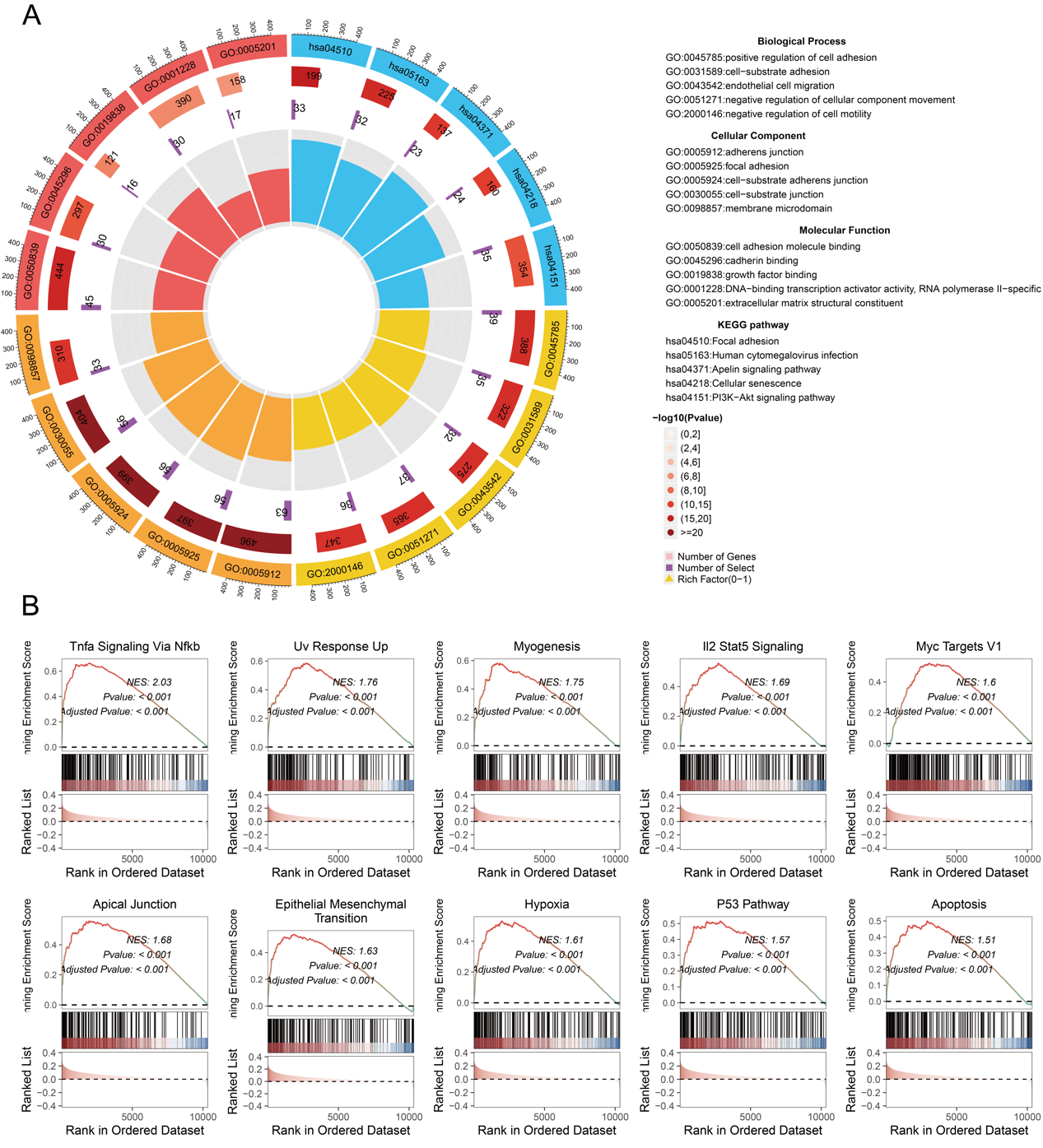


**Supplementary Figure.3** Functional enrichment analysis. **(A)**. GO/ KEGG analysis of differentially expressed genes. **(B)**. GSEA analysis of differentially expressed genes.


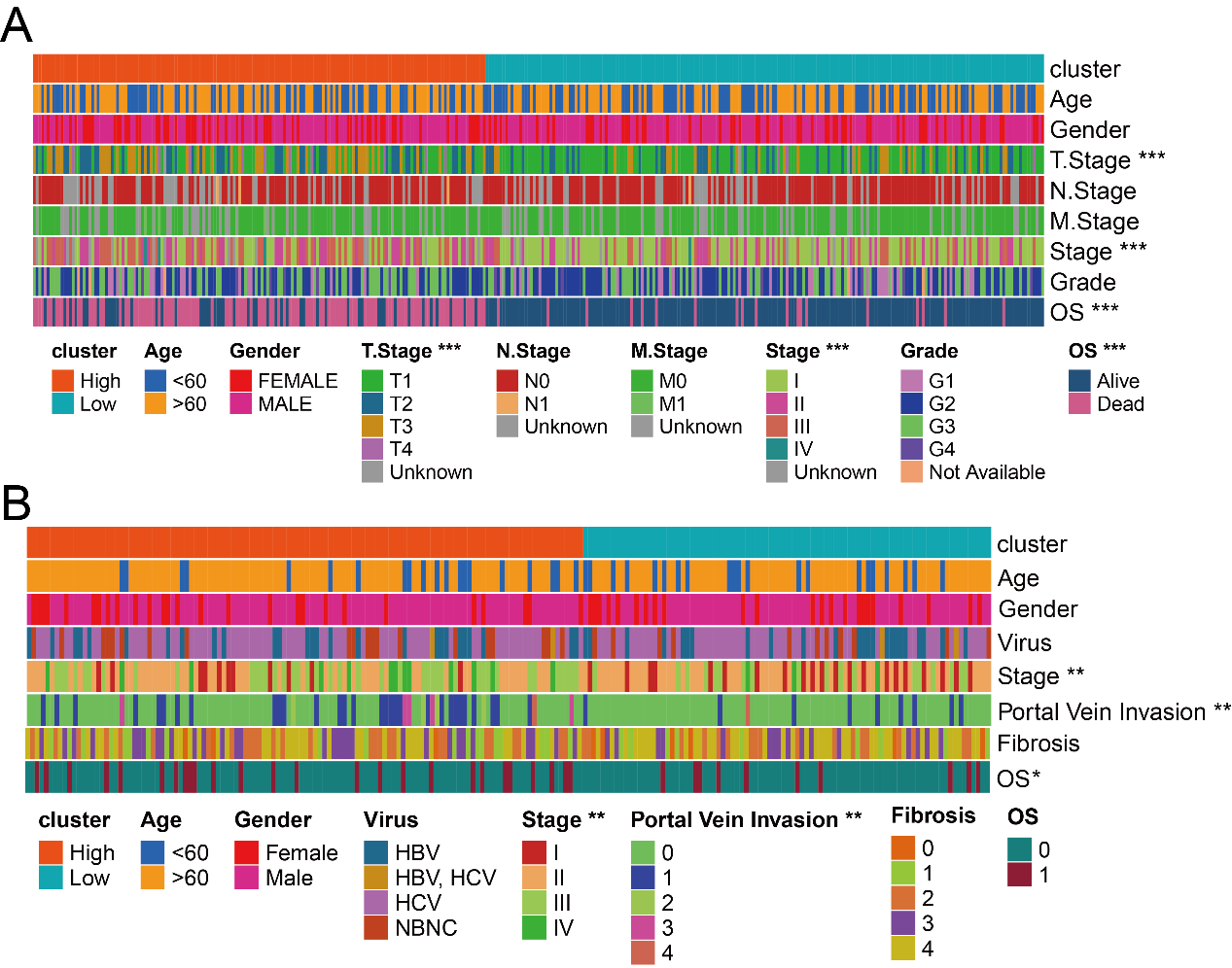


**Supplementary Figure.4** Distribution of clinical information in high and low-risk groups for the training and validation sets. **(A)**. In TCGA-LIHC, the high-risk group exhibits later T. stage and Stage, and worse clinical prognosis (p<0.001). **(B)**. In HCCDB18, the high-risk group presents later Stage and is more likely to have Portal Vein Invasion (p<0.01), with a worse prognosis (p<0.05). (* p<0.05, ** p<0.01, and *** p<0.001.)


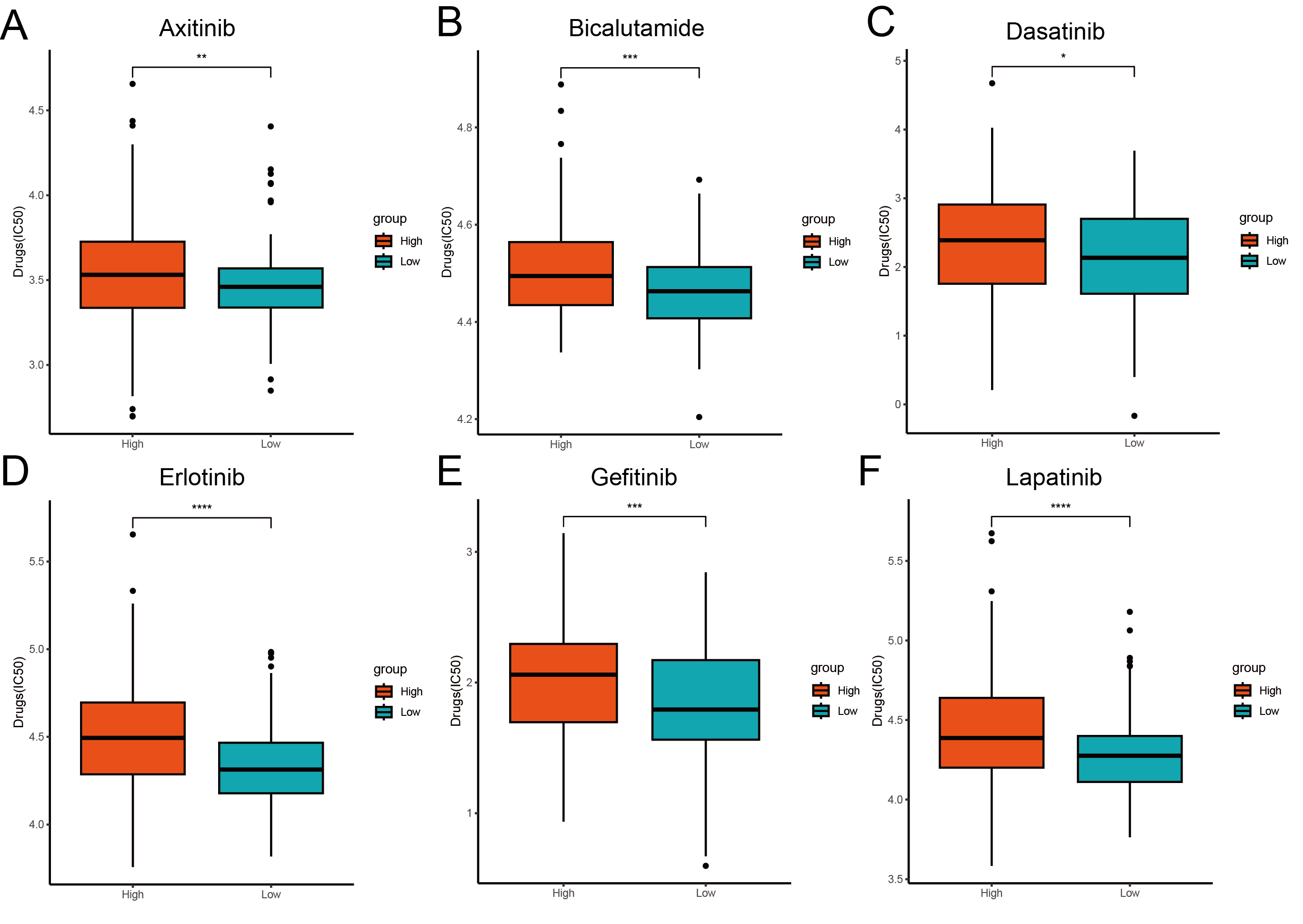


**Supplementary Figure.5** Drug prediction. The high-risk group demonstrates a poorer response to axitinib, bicalutamide, dasatinib, erlotinib, gefitinib, and lapatinib (p<0.05). (*p<0.05, ** p<0.01, *** p<0.001, **** p<0.0001.)


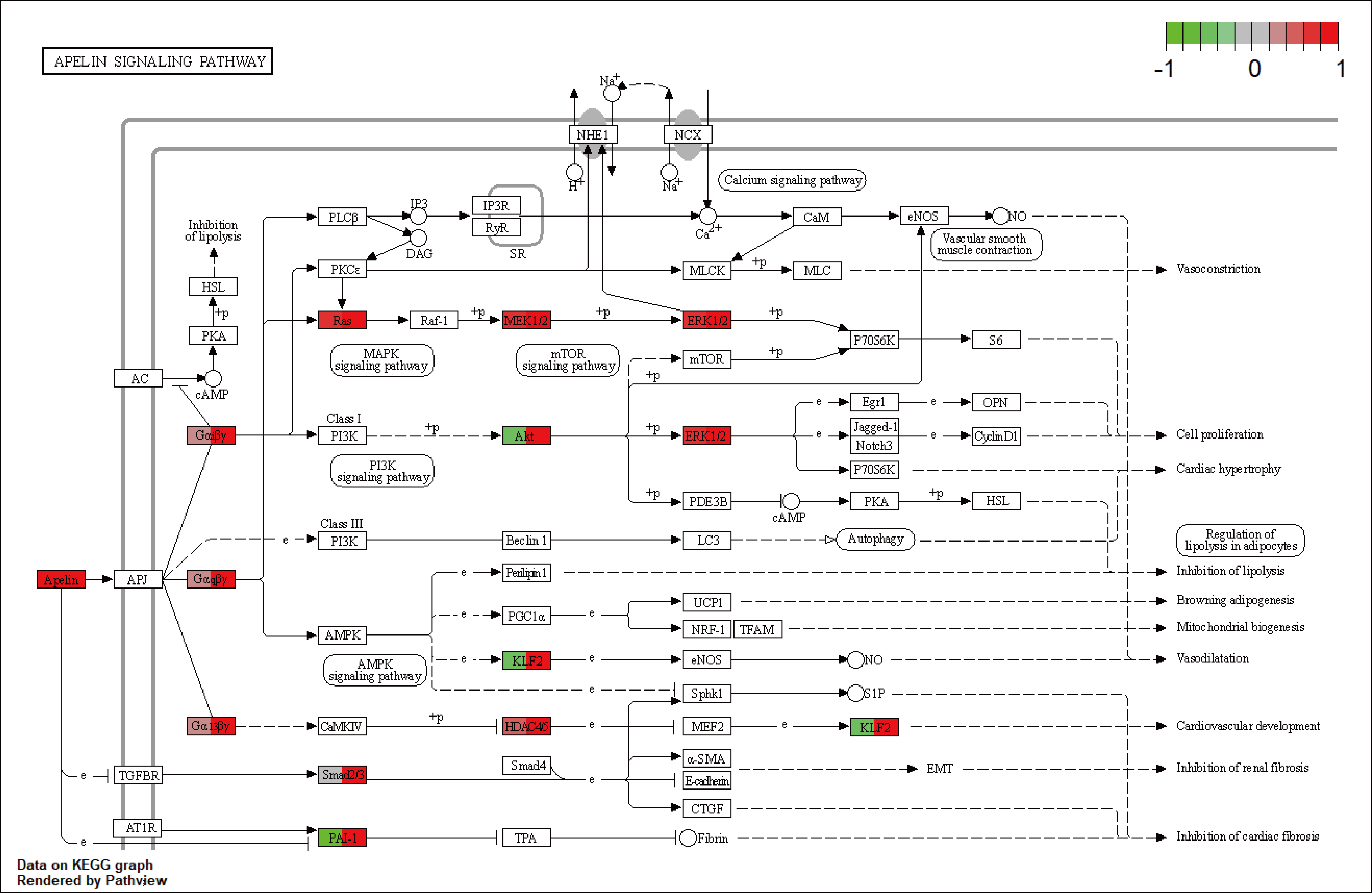


**Supplementary Figure.6** graphical illustration illustrating the function and pathway position of model genes within the apelin/APJ signaling pathway.
